# Changes in Posture and Interactive Behaviors as Infants Progress From Sitting to Walking: A Longitudinal Study

**DOI:** 10.3389/fpsyg.2019.00822

**Published:** 2019-04-12

**Authors:** Sabrina L. Thurman, Daniela Corbetta

**Affiliations:** ^1^Department of Psychology, Elon University, Elon, NC, United States; ^2^Department of Psychology, The University of Tennessee, Knoxville, Knoxville, TN, United States

**Keywords:** infancy, locomotion, posture, interactive behaviors, exploration, longitudinal study

## Abstract

This longitudinal study assessed how infants and mothers used different postures and modulated their interactions with their surroundings as the infants progressed from sitting to walking. Thirteen infants and their mothers were observed biweekly throughout this developmental period during 10 min laboratory free-play sessions. For every session, we tracked the range of postures mothers and infants produced (e.g., sitting, kneeling, and standing), we assessed the type of interactions they naturally engaged in (no interactions, passive involvement, fine motor manipulation, or gross motor activity), and documented all target transitions. During the crawling transition period, when infants used sitting postures, they engaged mainly in fine motor manipulations of targets and often maintained their activity on the same target. As infants became mobile, their rate of fine motor manipulation declined during sitting but increased while kneeling/squatting. During the walking transition, their interactions with targets became more passive, particularly when sitting and standing, but they also engaged in greater gross motor activity while continuing to use squatting/kneeling postures for fine motor manipulations. The walking period was also marked by an increase in target changes and more frequent posture changes during object interactions. Throughout this developmental period, mothers produced mainly no or passive activity during sitting, kneeling/squatting, and standing. As expected, during this developmental span, infants used their body in increasingly varied ways to explore and interact with their environment, but more importantly, progression in posture variations significantly altered how infants manually interacted with their surrounding world.

## Introduction

Infants develop curiosity about the world. This encourages them to interact and explore objects and people in it out of their own volition (Piaget, 1936). By acting on the environment with their own bodies, infants come to understand their surroundings, and learn the interrelationships between their own action capabilities and the features of the environment that support those actions (Gibson, 1988). Postures – the particular body and limb configurations used at any moment – mediate action development in meaningful ways (Rochat and Bullinger, 1994). For example, the acquisition of each new posture provides a unique lens through which infants can view the world, and it allows them to accrue a range of possibilities for moving about and physically interacting with the environment (e.g., Adolph, 2008; Pierce et al., 2009; Thurman and Corbetta, 2017). Such interactions contribute to psychological change (Campos et al., 2000), and lay a foundation for future cognitive skills (e.g., Bornstein et al., 2013; Libertus and Violi, 2016), and long-term brain development (Bernier et al., 2016).

Infants’ motor and interactive behaviors also often occur in an environment attended by their caregiver (e.g., Campos et al., 2000; Bigelow et al., 2004; Lobo and Galloway, 2012; Karasik et al., 2014; Fukuyama et al., 2015). The interactive activities that mother and child each produce in the environment may change as infants acquire new motor skills. Little research has described the posture and physical interaction patterns that mother and child display in free-play activities over the first 2 years of life. This study aims to capture how infants and their mothers use their bodies to manipulate targets in a playroom as the infants transition from sitting, to crawling, and walking.

### The Role of Posture in Infant Interaction and Exploration

Postures can be seen as a means through which infants use their bodies to interact with their surroundings. Depending on the motor skill level and posture used, physical interactions with objects can be facilitated or reduced. For example, when sitting, infants’ hands are free allowing them to manipulate and explore objects, sometimes in sophisticated ways (Rochat, 1989; Soska et al., 2010; Lobo and Galloway, 2013; Lobo et al., 2014; Soska and Adolph, 2014). When in prone, however, infants are limited to use one hand to lift their torso off the ground, while using the other to reach out for an object which can reduce the range of actions (Rocha and Tudella, 2008). When standing and walking, infants’ hands are free again and can further expand their range of possibilities, while during hands-and-knees crawling, infants are less likely than walking infants to carry objects (Karasik et al., 2012). Thus, each posture provides unique problem spaces and constrains how the body can be used (Adolph, 2008).

Postures can also alter the stability of the body, the demand of attentional resources, and what can be perceived in the surroundings (e.g., Kretch et al., 2014; Franchak et al., 2018). Certain postures and their relative stability can even influence the use of the limbs and hands. For example, transitioning from sitting to crawling, and from crawling to walking affects the way infants use their arms for reaching and retrieving objects (Corbetta and Bojczyk, 2002; Corbetta and Thelen, 2002). Unstable postures often require more of the infants’ effort for balance. For example, the hands are needed to hold onto surfaces during cruising (Berger et al., 2014), or they may be simply needed to balance the body when newly standing (Ledebt, 2000). Attention to balancing one’s posture may limit object manipulations, however, holding objects can help stabilize standing in infants (Claxton et al., 2013). Finally, energetically expensive forms of locomotion such as crawling can affect object interactions (Dosso and Boudreau, 2014).

Thus, research has shown that postural progression and postural control can modulate infant’s experiences with objects, people, and their wider environments. Furthermore, these posture-specific working spaces not only change depending on the posture adopted, but also may affect object interactions at any given moment, and over the course of motor development. In this study, we examine more closely how infants’ expanding repertoire of postural skills as they acquire locomotor skills, affects their manipulatory behaviors and interactive activities with objects in their surroundings. Prior research has provided single snapshots of infants’ interactive behaviors at certain developmental times. Here, we track how infant interactive behaviors reorganize as they progress from sitting through walking. In doing so, we also document mothers’ posture and interactive behaviors as their infants acquire new motor milestones.

### The Role of Locomotion in Infant Target-Directed Behavior

The emergence of self-produced locomotion elicits significant improvements in target-directed behaviors (e.g., Gustafson, 1984; Campos et al., 2000). Before infants can produce hands-and-knees crawling, they almost exclusively interact with objects, people, and contexts in their close proximity (Pierce, 2000; Zachry and Mitchell, 2012). With the onset of self-produced locomotion, however, infants become active agents in their own expanding world. Their interactions shift to the wider space, and they engage in more target-directed actions such as object manipulation (Gustafson, 1984). Zachry and Mitchell (2012), who observed infant behaviors in childcare centers, discovered that crawling infants displayed more goal-directed actions (e.g., activity initiation) than did pre-crawling infants. This expansion in interactions with objects was found to relate to increases in spatial exploration of their surroundings. In free-play, mother and infant travel more distance and spread their activities around a room more following the onset of crawling, but infants’ spatial explorations of the room broadens significantly more than that of their mothers’ (Thurman and Corbetta, 2017). Infants’ expanding spatial exploration patterns also strongly correlate to the number of bouts of interactions they perform in the room (Thurman and Corbetta, 2017). Clearly, the emergence of self-produced locomotion brings many changes in infants’ patterns of interactions with objects and target-directed behaviors.

Interestingly, walking infants’ behavior appears more deliberate and target-directed compared to crawling infants. Walkers will travel even further distances to obtain a desired toy or reach a destination compared to crawlers, whose interactions appear more opportunistic (Pierce et al., 2009; Dosso and Boudreau, 2014). They also become more interactive with toys and people in the room, compared to crawlers (Campos et al., 2000; Clearfield et al., 2008; Clearfield, 2011; Karasik et al., 2011).

These studies together highlight the role of forms of self-produced locomotion in infants’ expanding explorations of their surroundings. But, how does the acquisition and expanding repertoire of new postures relate to target interactions? The behavioral rhythm of infant action patterns has been characterized by frequent and abrupt movements (Reed, 1988), but we know little about how infants actually use their bodies as they transition between moments of target-interactions and moments of pause. To our knowledge, no study has longitudinally examined how pre-locomotor, crawling, and walking infants’ expanding postural skills modulate the way they interact with targets in their environment. We also are not aware of any studies investigating these patterns in mothers, although prior work has indicated that caregivers seem to mirror certain patterns of movement and postural behaviors to match those displayed by their infants (Thurman and Corbetta, 2017; Franchak et al., 2018). It remains unclear whether mothers’ physical interactive behaviors also change as their infants acquire new locomotor skills.

### The Current Study

This study is an extension of a previous report (Thurman and Corbetta, 2017) using the same longitudinal dataset. In that report, we delineated patterns of mother-infant spatial exploration, the number of object interaction bouts and posture changes displayed, and the proportion of time intervals infants and mothers spent in certain postures during free play. We found that over time, infants increased their interactive behaviors, traveled further, and spread their exploration of the room more widely than their mothers. These trends in spatial exploration were highly correlated with the number of posture changes infants and mothers performed, but interactive behavior and posture changes were positively correlated only in the infants. This seemed to suggest that infants used their postures for movement and discovery, whereas mothers seemed to play a more supportive role.

In this report, we aim to further address how the postures adopted in the moment, at different periods of development, affected the type of interactive behaviors performed on objects in the room. While in certain postures, were infants passively holding objects, finely manipulating them, or performing gross motor actions? We also wanted to capture how frequently infants transitioned between targets, if they had moments of no interaction, and whether infants changed their postures during target transitions during the session and as they progressed through developmental time. Active exploration of one’s surroundings is not limited to manipulating objects with the hands or traveling to different locations. It may also entail shifting posture while interacting with objects or furniture in the surrounding. From an embodiment perspective, using the body as a whole provides new opportunities for interaction and discovery, and can provide new means-end to explore, achieve novel activities, and learn about objects in the environment. If this is a correct assumption, we should find greater postural diversity when infants are engaging in targeted behaviors compared to non-targeted behaviors.

We tracked interactive and postural activities in 13 infants and their mothers as the infants acquired crawling and walking skills. Our sampling, spanning several months across the first 2 years of life, allowed us to investigate natural changes in these behaviors in both infants and mothers, to determine how they occurred in a less controlled environment, and most importantly, to address how physical interactive activities reorganized as infants developed expanding postural forms over time. We asked, do infants and mothers alike shift interactive behaviors as infants acquire locomotion? Do interactive behaviors depend on the posture performed in the moment? And, do transitions between targets occur while maintaining or changing posture?

During the *pre-locomotor* period, we expected that infants would demonstrate more fine motor manipulations of objects while sitting. Given their limited range of postural skills, their posture would not differ greatly between moments of interactions with targets and moments of no interactions. Also, when transitioning between targets, they would do so while maintaining the same posture.

During the *crawling* period, infants begin squatting and kneeling. Because these postures will be novel and somewhat unstable, we expect that infants will engage in more passive involvement with targets (e.g., holding, hands on) when in those postures. During this period, infants orient more to their wider surroundings (Campos et al., 2000). As a result, we expect that infants will engage in less fine motor manipulation when sitting, since sitting can now be used as a transition posture (Kretch et al., 2014; Soska et al., 2015). Crawling infants also have more postural options available. These postural options may be more widely used when engaged with targets, compared to when not engaged with targets, or they may be used when transitioning from one target to another.

Finally, during the *walking* period, as infants have gained more postural experience and stability in squatting/kneeling postures, we expect that they may rely less on sitting postures for fine and gross motor activities, but increase their reliance on squatting/kneeling postures for these interactive behaviors. Furthermore, as infants now spend more time standing upright, they may display more passive involvement with objects when in that posture, as this newly acquired and unpracticed posture places more demands on balance. During this period, infants may begin to show an even wider range of postures when interacting with targets compared to when they are not interacting with targets. We also expect continued shifts in posture during transitions from one target to another.

We anticipate that mothers will not display significant changes in their use of postures for interaction over time. Particularly, as their infants gain more autonomy and become able to transition between targets more independently, mothers may take a more laid-back role with a decrease in interactive behaviors.

## Materials and Methods

### Participants

Participants in this study were the same as in Thurman and Corbetta (2017). Thirteen firstborn infants (6 females) and their mothers were followed every other week in our laboratory from the time infants were 6 months of age (*M* = 6.0, *SD* = 0.3 at first session), until they had 2 months of walking experience (*M* = 14.9, *SD* = 1.2 at final session). Visits totaled 247 sessions across all participants. There was no attrition. Table 1 reports the number of sessions each dyad contributed to the study.

**Table 1:** Number of sessions contributed by each dyad during the whole study.

Dyad ID	# of study sessions contributed	# of sessions used in analyses			Age of onset (months)	
			
		Pre-crawling	Crawling	Walking	Crawling	Walking
1	19	5	6	5	9.3	12.4
2	19	5	8	5	8.3	12.6
3	18	5	6	5	8.8	11.8
4	24	5	8	5	10.9	14.9
5	17	3	6	5	7.2	10.2
6	21	5	8	5	10.0	13.9
7	17	4	7	5	7.9	11.5
8	17	0	7	5	6.1	9.4
9	23	5	9	5	10.3	15.1
10	18	5	6	5	9.5	12.5
11	18	5	6	5	10.1	12.8
12	17	4	7	5	7.8	11.4
13	19	5	9	5	8.3	13.1


*Number of sessions used for the analyses ranging from 5 sessions (when available) before crawling onset up to 5 sessions following walking onset. The infant ages for crawling and walking onsets according to Touwen’s (1976) Group III Neurological Assessment Scale are also reported.*

All participants were recruited from a human subject database maintained by the Child Development Research Group at the University of Tennessee. We sent invitations for participation by mail when infants were around 5 months old. Interested families were invited to attend a non-committal informational session in our laboratory before deciding to participate. Thirteen out of 14 families who attended the information session decided to be a part of the study and signed informed consent forms. All infants were healthy throughout the duration of the study and were free of physical impairments. All families were non-Hispanic White, and over half fell into categories consistent with middle socioeconomic status (e.g., college degree(s), middle-income households). At each session, families were compensated $10, and at the end of the study, were given a certificate of completion, a photo book with photographs and milestones from each session, and copies of all DVD recordings.

### Materials

#### Room and Toys

**FIGURE 1:**
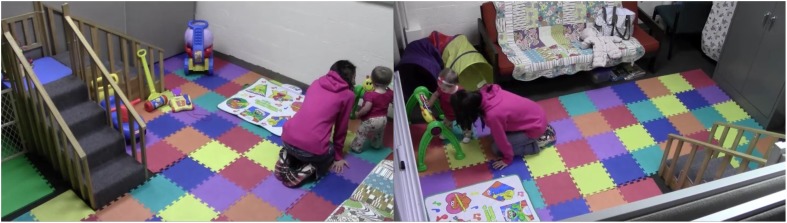
Synchronized camera views of laboratory free play space during a mother and infant free-play interaction.

Infants participated in 10 min free-play sessions with their mothers held in a brightly lit, temperature controlled laboratory space. The free-play room measured 3.3 m × 3.7 m, the size of a standard bedroom. The space was accessed through a small walkway that could be closed off with a baby gate at the mother’s request. The room contained a couch against one wall, and a small set of infant-sized stairs (54 cm tall), located directly across from the couch, and a large metal cabinet, a chair, and a bookshelf against another wall. Colorful foam tiles covered most of the floor and posters were placed on the walls around the room (see Figure 1). In addition to these room features and large furniture items, the room was equipped with a variety of gender-neutral colorful objects to elicit fine and/or gross motor exploration (e.g., the pull-string toy resembling a traditional phone could be rolled across the floor or manually manipulated by pushing buttons). Table 2 lists all possible targets present and available in the playroom, including the mother and infant. As shown, the room contained at all times between 28 and 29 possible targets (this includes the furniture, walls, and flooring; sometimes the mothers used their own items). Of those items, 23 (82%) were always present in the room for the duration of the entire study. Five objects better suited for younger infants were switched out at some point during the study depending on infants’ locomotor skill progression, and six new ones were added (e.g., the sit-on rocking horse was replaced with the rolling melody push toy).

**Table 2:** List of all targets used in study, their classifications, and the length of time they were available in the playroom.

Target always present in the playroom	Target classification
Small set of infant-sized stairs	Furniture/room
Couch	Furniture/room
Wall	Furniture/room
Green chair	Furniture/room
Bookshelf	Furniture/room
Large metal cabinet	Furniture/room
Letter-shaped magnets	Object
Wheeled sit-on push cart with a handle (43 cm tall at handle)	Object
Caterpillar-themed musical activity center with adjustable legs, spinners, buttons, and rattles (52 cm tall)	Object
Books	Object
Magazines	Object
Bead maze	Object
Box toys – box and lid	Object
Box toys – plush rattle with plastic rings	Object
Box toys – plastic seahorse-shaped rattle	Object
Box toys – hand-held toy with multiple handles, buttons, spinners, and shakers	Object
Box toys – set of three small balls that could easily fit into an infant’s hand	Object
Box toys – musical tiger toy with buttons	Object
Box toys – musical bear toy with buttons	Object
Foam tile flooring (only coded as a target if removed from floor)	Object
Foam seat	Object
Mother	Other person
Infant	Other person
**TARGETS REMOVED AT SOME POINT IN THE STUDY**	
Plush jumbo block with different activities on each side (e.g., zipper, mirror, squeaker, vibrating plush ball on a pull string)	Object
Sit-in circular activity center with rattles, a spinner, buttons, and a teether (43 cm × 61 cm)	Object
Round activity table with interchangeable top pieces with buttons and sliders (41 cm × 36 cm high)	Object
Sit-on rocking horse with handles on each side	Object
Bumbo seat (only coded as a target for mothers)	Object
**TARGETS ADDED LATER IN THE STUDY**	
Mat that produced music when pressure was applied	Object
Large rubber bounce ball (28 cm wide)	Object
Flexible and collapsible nylon tunnel (48 cm × 185 cm)	Object
Rolling melody push toy with a handle	Object
Wagon with a handle and basin that could be raised (29 cm tall)	Object
Small musical rolling pull-string toy resembling a traditional phone	Object
**OTHER**	
Personal item brought by mothers (e.g., cell phone, car keys, diaper bag, etc.)	Object


Sessions were recorded with two Canon Vixia HFR32 digital video cameras that were positioned on opposite sides of the room. Together, the two camera views captured all activity in the room.

#### Assessment of Postural Control

Touwen’s Group III Neurological scale is an infant assessment technique designed for the evaluation of posture, muscle tone regulation, reflexes and reactions, trunk coordination, and fine and gross motor coordination (Touwen, 1976). The technique has good reliability and validity assessment scores and takes about 15 min to administer (Heineman and Hadders-Algra, 2008; Hadders-Algra et al., 2010). The assessment was administered at the end of each session in the presence of the parents.

For the purposes of the current analyses, we used two items from this scale: locomotion in prone position (crawling), and walking. *Locomotion in prone position* was assigned the following scores: 0 to indicate no change in spatial position, 1 for wriggling or pivoting movements, 2 for abdominal progression using the arms only, 3 for abdominal progression using the arms and legs, 4 for progression by way of a mixed pattern of abdominal progression using the arms and legs and hands-and-knees crawling, and 5 for hands-and-knees crawling. We used the score of 5 as a cutoff point between pre- and post-crawling. *For walking*, the scores were: 0 for unable to walk, 1 for walking when held by both hands, 2 for walking when held by one hand, 3 for walking a few (less than seven) independent steps, and 4 for walking at least seven independent steps. We used the score of 4 as the cutoff point between pre- and post-walking. Consistency of these cutoff scores, when infants first exhibited those locomotor skills in the laboratory, were reassessed in subsequent sessions. Table 1 reports the ages at which those milestones were attained.

### Procedure

Before each session, objects were positioned in consistent locations in the room (e.g., the sit-on pushcart was always placed on the floor in the bend of the stairs), but all objects could all be moved freely around the room by the participants except for the furniture.

At their arrival, dyads were given time to settle into the laboratory space. An experimenter turned on both cameras and bounced a small rubber ball in the center of the room to provide an event that could be easily identified in both recordings for later video synchronization. There were three 10 min conditions, which were randomized across sessions and dyads. In one condition, mothers were given a problem-solving toy (e.g., fit-the-shape toy). Another condition involved a startle toy (e.g., jack-in-the-box), and the third condition was the free-play. Only data from the free-play session is included in the current report.

During the free-play condition, mothers were asked to play with their infants as they normally would. An experimenter monitored each session from an adjacent location that was not visible to the participants. Out of the 247 recordings, only 14 were paused at the mother’s request for diaper changes, feedings, dealing with fussiness, etc. until the mother indicated that the session could resume.

### Coding and Dependent Measures

Session recordings from both video cameras were imported into The Observer XT and synchronized for behavioral coding (see Figure 1 for view from both cameras). We used a time sampling of 15 s intervals to capture general trends in these behaviors over the first 2 years. At each 15 s interval across the 10 min free-play session, we coded infants’ and mothers’ postures, and the types of physical interactive behaviors they produced with targets. To provide coders with sufficient information to accurately code behaviors, we used 2 s of video prior to each 15 s interval to interpret the behavior (e.g., standing vs. walking). Thus, behaviors occurring between 6:58.0 and 7:00.0 would be examined for coding the interval at 7:00.0. The total corpus corresponded to 41 h of video recordings and 9,880 15 s intervals of free play. Codes are explained below.

#### Posture

We adapted the posture coding scheme used by Thurman and Corbetta (2017). This coding scheme delineates nine posture categories from a range of positions and movements. Posture categories were as follows: *being repositioned, held* or *carried* by the mother*, laying down, sitting, stationary on all fours, kneeling*/*squatting, crawling, standing, cruising*, and *stepping*. The posture displayed at each 15 s interval in the session was coded. If the participants’ body was not fully visible during an interval, that interval was excluded from the analyses, but this represented on average less than 1% of the overall percentage of infants’ intervals (Mean = 0.92%, *SD* = 0.83%, range = 0.00–2.45%).

#### Interactions With Targets

We considered whether participants were directly and actively engaging/interacting with a target in a physical way (meaning they were in direct contact with the target). Targets included toys, furniture, or the other person. Instances when participants were not contacting a target were coded as “nothing” (e.g., an infant simply sitting on the floor and looking at the mother). We derived a few variables from this coding. First, we counted how many intervals in each session participants physically interacted with a target vs. not. From this, we derived the proportion of intervals that participants spent in targeted vs. untargeted behavior.

We also derived information about how targets changed across successive intervals. We classified five types of target transitions. *Target-to-same-target transitions* occurred when participants continued to interact with one target from one interval to the next (e.g., climbing the stairs in one interval to pulling up on the stairs in the next interval). *Target-to-new-target transitions* occurred when participants switched from one target to a different target from one interval to the next (e.g., bouncing the ball, then patting the mother). *Target-to-nothing transitions* occurred when participants engaged with a target in one interval, but then did not in the next (e.g., hand on the stairs, then simply sitting on the floor). *Nothing-to-target transitions* occurred when participants went from not interacting with a target in one interval to engaging with a target in the following interval (e.g., standing on the floor, then climbing on the couch). Finally, we coded *nothing-to-nothing transitions* if participants went from one interval to the next and did not interact with a target in either interval (e.g., sitting on the floor, then laying down). We counted how many times each of the five target transitions occurred in each session, then normalized the counts out of the total number of interval-to-interval transitions possible in each session. Using the posture coding described above, we also derived whether infants simultaneously changed or maintained their postures during target transitions.

##### Interactive behaviors

Interactive behaviors with targets were coded for each interval based on four categories. *No activity* indicated that the participant was not physically interacting with a target (e.g., standing in the middle of the floor). Participants also could engage in *passive* and/or *minimal involvement* (e.g., hand on a toy, sitting stationary on the cart). More involved complex movements were classified as either *general fine motor manipulation* (e.g., pressing buttons, spinning), or *general gross motor activity* (e.g., climbing on the couch, pushing wagon, throwing ball) which corresponded to all behaviors not involving fine motor manipulation. We investigated these interactive behaviors during intervals in which participants were either sitting, squatting/kneeling, or standing because those were the three most commonly displayed postures (Thurman and Corbetta, 2017). For each session, we counted how many intervals of sitting, squatting/kneeling, and standing postures corresponded to each category of interactive behavior, and then derived the proportion of intervals participants engaged in each type of interactive behavior while in each of those postures.

### Inter-Rater Reliability

Pairs of trained coders independently coded between 20 and 23% of the data depending on the analyses. Video segments were selected randomly throughout the entire developmental period and across dyads. For the infants, Kappa’s agreements (and interrater correlations) were 0.73 (*r* = 0.91) for posture, 0.94 (*r* = 0.81) for targets, and 0.84 (*r* = 0.74) for interactive behaviors. For the mothers, Kappa’s agreements (and interrater correlations) for these codes were 0.89 (*r* = 0.81), 0.92 (*r* = 0.90), and 0.86 (*r* = 0.80), respectively. Disagreements on these reliability sessions were resolved through discussion.

### Analyses

Infants in our study learned to crawl and walk at different times and therefore were followed for different lengths of time. To structure our analyses, we included data from 5 sessions prior to crawling onset up to 5 sessions following walking onset (see Table 1). For some analyses, we used Pearson correlations on each infant’s and mother’s data over this entire period from sitting to walking to test developmental trends independent of the number of sessions each infant received. Pearson was chosen because it fits a linear trend on the data points while maintaining the developmental order of the sessions (Spearman ranks orders the data, hence potentially altering the developmental order). The individual correlation values obtained were then used with the non-parametric Friedman test to compare general developmental trends between variables. If the Friedman test yielded significant differences (*p* 2-tailed), we performed Wilcoxon tests (*p* 2-tailed) to determine where the differences lied.

We further analyzed the developmental changes in infants’ and mothers’ interaction patterns around the onset of hands-and-knees crawling and upright locomotion by running Generalized Estimating Equations (GEE) with a Bonferroni correction for multiple comparisons on segments of data covering 5 sessions prior and 5 sessions following the onsets of those locomotor skills. GEEs are particularly adequate for longitudinal data because they take into account the dependency and ordering of the data within subjects in repeated measures. GEEs assessed differences between mothers and infants, determined which behaviors were produced significantly more, and whether they changed as a function of sessions. Because infants had between 6 and 9 crawling sessions before walking onset, some sessions were used for the computation of the 5 post-crawling sessions and for the 5 pre-walking sessions. As a result, we did not run statistical tests to assess changes between post-crawling and pre-walking sessions.

## Results

### Postural Skills Progression

Infants sat independently at an average of 6.6 months (*SD* = 0.6), crawled on all fours at 8.8 months (*SD* = 1.4), stood independently at 11.2 months (*SD* = 1.2), and walked at least seven paces at 12.4 months (*SD* = 1.6). Four infants could sit independently for at least 30 s at their first session in the study, and one infant (ID#8, Table 1) crawled on all fours.

### Targeted Behavior

We first examined to which extent infants’ and mothers’ each engaged in target-directed behaviors. A GEE on the percent of intervals in targeted behavior around the crawling period, using dyad (mothers vs. infants) and session (10) as predictors revealed a main effect of dyad, and a dyad × session interaction. On average, infants engaged in targeted behaviors significantly more (79.47%) than their mothers [60.64%, Wald χ^2^(1) = 33.108, *p* < 0.0001]. Furthermore, over the 10 sessions, infants’ targeted behaviors increased up to 85.02%, while mothers only increased to 63.30% [Wald χ^2^(9) = 18.349, *p* < 0.031].

During the walking transition, the GEE analysis using the same predictors, similarly, revealed a main effect of dyad, and a dyad × session interaction. Again, infants displayed on average a higher percentage of targeted behaviors (85.37%) than their mothers [56.69%, Wald χ^2^(1) = 9.698, *p* < 0.0001]. Over the 10 sessions, infants’ targeted behaviors increased on average from 80.99 to 85.33%, while mothers’ decreased from 63.87 to 56.60% [Wald χ^2^(9) = 23.885, *p* < 0.004].

### Number of Posture Configurations Displayed

**FIGURE 2:**
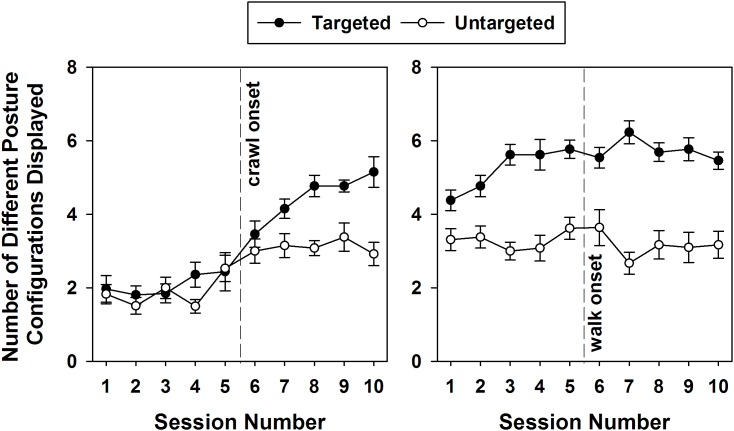
Mean numbers and standard errors of posture configurations displayed by infants over the 10 sessions surrounding the crawling (left) and walking (right) transitions as function of targeted engagement.

As infants developed mobility, they also widened their range of posture use, but they did so mainly during targeted interactions. Figure 2 represents the number of different posture configurations (not the number of posture changes) infants displayed over the 10 sessions around crawling and walking onsets during intervals of targeted or untargeted behavior. Over the crawling transition period, a GEE ran on this variable using behavior (targeted vs. untargeted) and session as predictors confirmed a main effect of both. Figure 2 shows that, on average, infants displayed more varied posture configurations during intervals of targeted (3.02) than untargeted behavior [2.39, Wald χ^2^(1) = 4.185, *p* < 0.041]. Further, the number of posture configurations displayed increased significantly over the 10 sessions [Wald χ^2^(9) = 40.497, *p* < 0.0001].

Around the emergence of walking, the GEE with the same predictors revealed a main effect of behavior, but not of session. During this transition period, infants displayed an average of 5.46 different postures configurations during intervals of targeted behavior compared to 3.20 in untargeted ones [Wald χ^2^(1) = 60.231, *p* < 0.0001].

**FIGURE 3:**
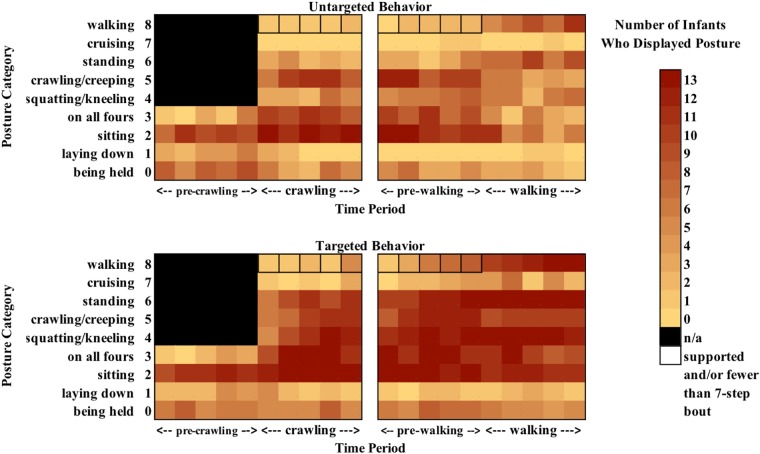
Heat maps representing the number of infants who displayed a given posture during a given session when engaging in untargeted (top) vs. targeted (bottom) behavior around the transition to crawling (left) and walking (right). Lighter colors indicate that fewer infants displayed a given posture.

The different types of posture infants displayed during each 10-session period surrounding the onset of crawling and walking are reported in Figure 3 by targeted behavior. The colors correspond to the number of infants that displayed each of the postures listed by session. This figure illustrates that between the first and second 10-session period, increasingly more infants diversified the range of postures they used to interact with their environment, but they did so mainly while actively engaging with targets.

**FIGURE 4:**
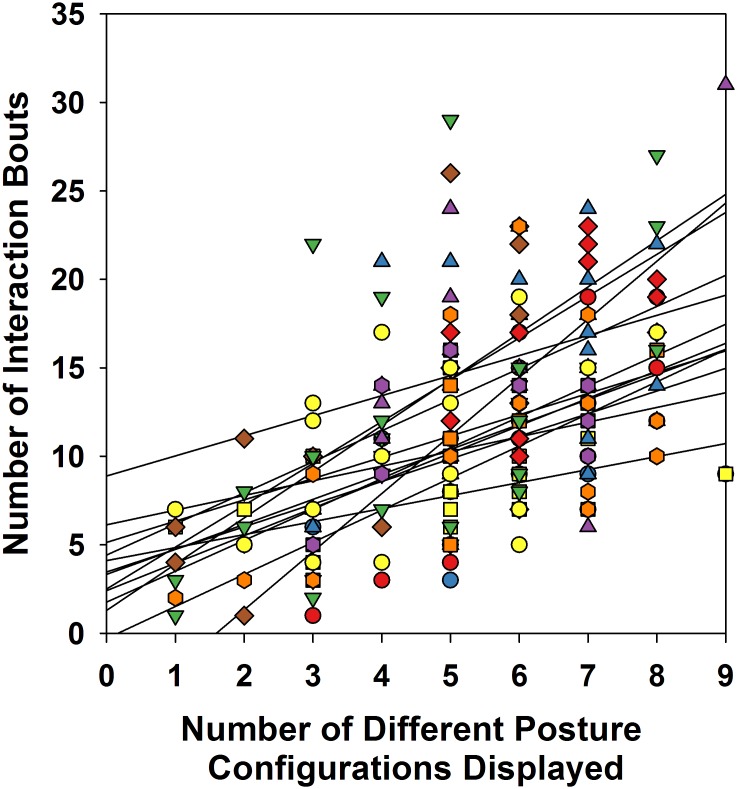
Pearson correlations and corresponding regression lines for each of the 13 infants, fitting the relation between the number of posture categories and frequency of targeted behaviors they displayed at each session. Each symbol type represents a different infant. Note that several symbols can line up over a same number of postural configurations. This occurs when infants produce identical numbers of postural configurations across several sessions, however, each with a different number of targeted interactions.

To verify whether posture diversity related to the frequency of targeted behaviors, we ran Pearson correlations on each infants’ data, pairing their number of posture configurations with their number of targeted behaviors by session (infants provided between 12 and 19 sessions for each correlation, see Table 1). All correlations were positive (see Figure 4). Nine out of the 13 infants’ correlations were significant above the 0.05 level (range: *r* = 0.284, *p* < 0.239 to *r* = 0.751, *p* < 0.001). For the majority of infants, as postural diversity increased, so did the frequency of targeted behaviors.

### Interactive Behaviors in Postures

Did interactive behaviors change as infants transitioned from sitting to walking and developed new postural forms? We focused on three postures most commonly produced (sitting, kneeling/squatting, and standing) and examined the types of interactive behaviors infants and mothers each produced when in those postures within and across sessions.

#### While Sitting

Sitting was the only posture performed throughout the entire study. We first ran an analysis on the entire data span to test the overall developmental trends. Then, we ran analyses by 10-session periods to focus more closely on the changes occurring around the crawling and walking transitions.

**FIGURE 5:**
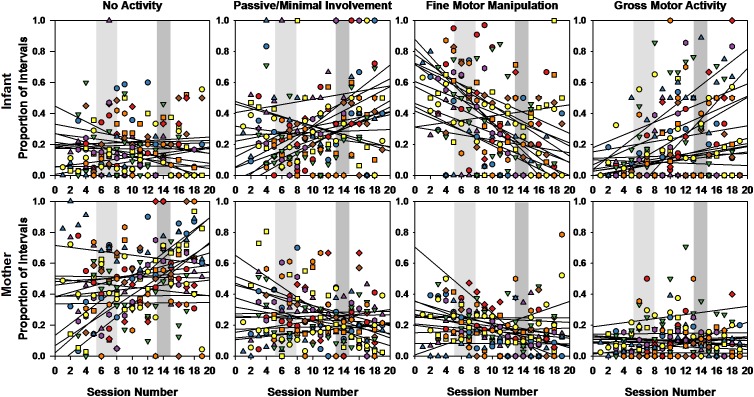
Pearson correlations and corresponding regression lines showing the overall developmental trends for all 13 dyads (infants top, mothers bottom) for the proportion of intervals spent engaging in each type of interactive behavior (left to right: no activity, passive/minimal involvement, fine motor manipulation, and gross motor activity) while sitting. Regression lines for each participant fit the proportion of intervals of a given interactive behavior from 5 sessions before crawling onset, up to 5 sessions after walking onset. Light gray and darker gray shaded areas span the range of session numbers during which different infants learned to crawl and walk, respectively.

Figure 5 displays the developmental trends for four types of interactive behaviors while infants and mothers were in sitting postures. Each regression line from the Pearson’s correlations corresponds to each of the 13 infant or mother. Friedman tests comparing the correlation values of those trend lines by interactive behavior for the infants and mothers separately revealed significant differences [infants: χ^2^(3) = 21.277, *p* < 0.0001; mothers: χ^2^(3) = 8.723, *p* < 0.033]. For the infants, the near zero correlations for no activity (mean *r* = -0.093) were significantly different from both the negative correlations in fine motor manipulation (mean *r* = -0.469; *Z* = -2.900, *p* < 0.004), and the positive correlations in gross motor activity (mean *r* = 0.316; *Z* = -2.760, *p* < 0.006). The negative correlations in fine motor manipulation were also significantly different from both the positive correlations in passive/minimal involvement (*Z* = -2.830, *p* < 0.005), and gross motor activity (*Z* = -3.180, *p* < 0.001). Thus, when sitting, over the study period, infants decreased the proportion of intervals they engaged in fine motor manipulation, they increased intervals in passive/minimal involvement and gross motor activity, while intervals in no activity remained about the same.

For the mothers, differences in correlation trends for interactive behaviors during sitting intervals were significant only between fine motor manipulations (mean *r* = -0.271) and both no activity (mean *r* = 0.255; *Z* = -2.341, *p* < 0.019) and gross motor activity (mean *r* = 0.030; *Z* = -2.132, *p* < 0.033). Mothers also displayed a decline in fine motor manipulations over developmental time, while increasing no activity and maintaining gross motor activity.

GEE analyses using dyad (infant vs. mother), interactive behavior, and session as predictors allowed us to assess more finely differences between the interactive behaviors displayed during sitting intervals, and capture infants/mothers differences at those transition times. Because our data were normalized within postures, for each GEE, we focused on the three interactive behaviors that displayed the largest developmental changes over the 10-session period as our first selection criterion, and then, we used the mostly represented behavior as our second criterion based on the combined data from the infants and mothers.

**FIGURE 6:**
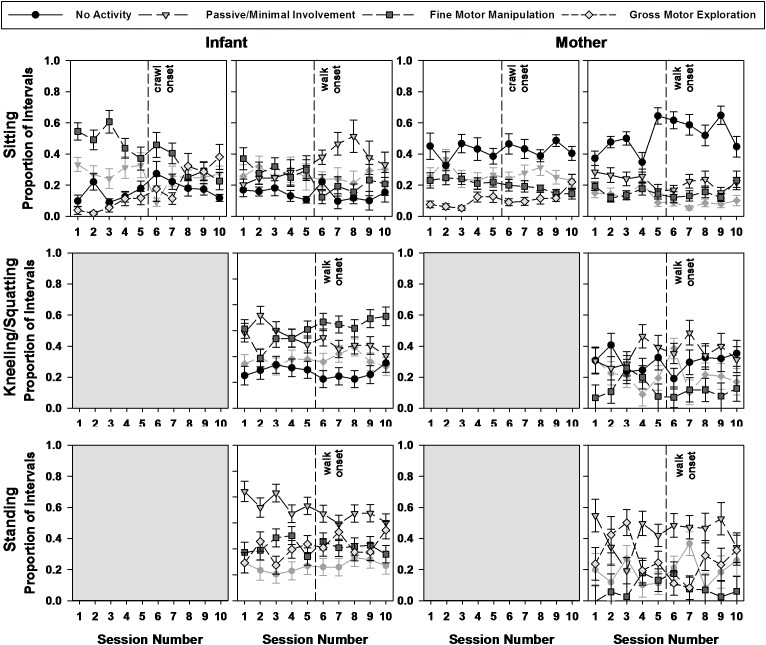
Mean proportions and standard errors of infants’ and mothers’ interactive behaviors by posture, by type of interactive behavior, by session number across the transition to crawling and walking, and by dyad member. The vertical lines on the graphs indicate the onsets of crawling and walking, respectively. The lines that are grayed out were not entered in the GEE analyses but are still plotted for illustration purposes.

*During the transition to crawling* (Figure 6, top), a GEE ran on the percent intervals of interactive behaviors performed in sitting using dyad (infant vs. mother), interactive behavior (fine manipulation, gross motor activity, and no activity) and session as predictors revealed a significant main effect of interactive behavior [Wald χ^2^(2) = 69.594, *p* < 0.0001]. Pairwise comparisons revealed that, as a whole, the average proportion of intervals of gross motor activity while in sitting was significantly lower (13.58%) than for no activity (29.60%) and fine manipulation (30.55%, all *p*s < 0.0001). A dyad × interactive behavior interaction [Wald χ^2^(2) = 108.658, *p* < 0.0001] indicated that the infants used on average 40.89% of their sitting intervals engaging in fine motor manipulations, while the mothers used an average of 42.35% of their sitting intervals in no activity. Further, an interactive behavior × session interaction [Wald χ^2^(18) = 69.384, *p* < 0.0001] revealed that intervals of fine motor manipulation while in sitting decreased over this 10-session period, while intervals of gross motor activity increased during this same period. Finally, a 3-way significant interaction of dyad × interactive behavior × session [Wald χ^2^(18) = 36.421, *p* < 0.006] indicated that the observed decline in fine motor manipulation and increase in gross motor activity while sitting were more pronounced for the infants than for their mothers.

*During the transition to walking*, a similar GEE using no activity, fine manipulation, and passive engagement as the three selected interactive behaviors revealed again a significant main effect of interactive behaviors [Wald χ^2^(2) = 31.865, *p* < 0.0001], a dyad × interactive behavior interaction [Wald χ^2^(2) = 143.834, *p* < 0.0001], an interactive behavior × session interaction [Wald χ^2^(18) = 39.030, *p* < 0.003], and a dyad × interactive behavior × session interaction [Wald χ^2^(18) = 52.345, *p* < 0.0001]. Pairwise comparisons indicated that during this period the average proportion of intervals of fine motor manipulation had now become overall significantly lower (19.84%) than the average proportion of intervals of no activity in sitting (33.02%) and passive involvement (27.79%, all *p*s < 0.001). The mothers and infants, however, differed greatly in their respective distribution of interactive activities. Mothers continued to spend on average a high percentage of their sitting intervals in no activity (51.64%), while infants only spent 14.38% in no activity (*p* < 0.0001). Further, during this period, infants, on average, used most of their sitting intervals for passive involvement (33.48%), and much less performing fine motor manipulations (24.41%) compared to the previous crawling period. In fact, the significant interactive behavior × session interaction over this walking transition, revealed that infants continued to decrease their rate of fine motor manipulation during sitting intervals over the 10-session period, while they increased their rate of passive involvement. Mothers further increased their rate of no activity while decreasing their rate of passive involvement during sitting intervals.

In sum, during the crawling transition, infants’ fine motor manipulation – which was their most frequent activity during sitting intervals – declined progressively, while their gross motor activity increased. During the transition to walking, infants’ fine motor manipulations during sitting intervals further declined, but now intervals of passive involvement increased. The mothers, when sitting, performed mainly no activity throughout the study period. Over time, they decreased their rate of intervals of all other forms activities.

#### While Kneeling/Squatting

Kneeling and squatting postures began to appear in the behavioral repertoire of the infants after they began to crawl, thus we examined manipulations in those postures only around the transition to walking (see Figure 6, middle). GEE analyses on the interval percentage of interactive behaviors performed in kneeling/squatting using dyad (infant vs. mother), interactive behaviors (fine manipulation, passive involvement, no activity), and sessions as predictors revealed a main effect of interactive behaviors [Wald χ^2^(2) = 34.257, *p* < 0.0001]. During kneeling/squatting intervals, infants and mothers on average engaged more in passive/minimal involvement (34.68%), than fine motor manipulation (26.37%) and no activity (19.06%, all *p*s < 0.019). However, a dyad × interactive behavior interaction [Wald χ^2^(2) = 88.959, *p* < 0.0001] revealed that infants performed on average more fine motor manipulation (40.31%) and passive involvement (33.38%) than no activity (7.94%), all *p*s < 0.0001), while mothers, during kneeling/squatting intervals, engaged on average more in no activity (30.18%) and passive involvement (35.97%), than fine motor manipulation (12.44%, both *p*s < 0.0001). A 3-way dyad × interactive behavior × session interaction [Wald χ^2^(18) = 31.718, *p* < 0.024] further identified that while infants’ kneeling/squatting intervals showed a decrease in passive involvement and increase in fine motor manipulation during the transition to walking, mothers displayed an increase in passive involvement.

Thus, during the walking transition period, infants’ fine motor manipulations occurred mainly during kneeling/squatting intervals, and not so much during sitting intervals. Mothers continued to maintain a relatively high level of no activity or minimal/passive involvement even when in kneeling/squatting.

#### While Standing

Around the transition to walking, infants also learned to stand (Figure 6, bottom). A GEE analysis on the percent intervals of interactive behaviors performed in standing using dyad (infant vs. mother), interactive behaviors (passive involvement, fine and gross motor activity), and sessions as predictors revealed again a main effect of interactive behaviors [Wald χ^2^(2) = 101.988, *p* < 0.0001]. The proportion of intervals of passive interactions during standing were on average higher (46.36%) than those for fine motor manipulation (14.85%) and gross motor activity (23.73%; all *p*s < 0.0001). However, a dyad × interactive behavior [Wald χ^2^(2) = 9.147, *p* < 0.010] revealed that while both mothers and infants produced on average high rates of passive behaviors during standing intervals (mothers = 42.52%; infants = 50.20%), they differed in their rates of fine motor manipulations. Infants produced on average 21.63% of fine motor manipulations intervals compared to 8.07% for the mothers (*p* < 0.043). Finally, a 3-way dyad × interactive behavior × session interaction [Wald χ^2^(18) = 46.669, *p* < 0.0001] indicated in infants a decrease in passive involvement and an increase in gross motor activity during standing, while no clear developmental trend reflected changes in the mothers’ interactive behaviors over those 10 sessions.

Together, these results suggest that passive involvement with targets, mainly performed during standing intervals around the transition to walking, decreased over the sessions as gross motor activities increased.

### Transitions Between Targets

**FIGURE 7:**
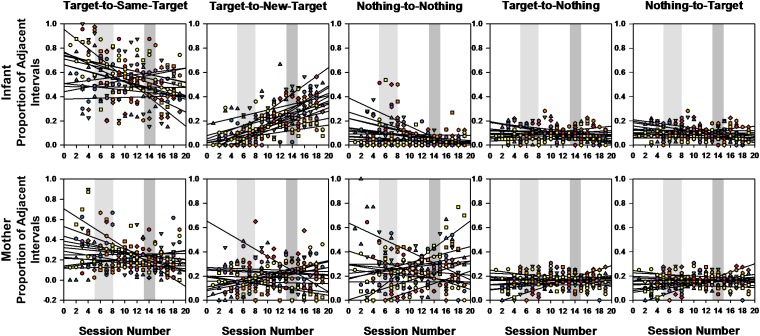
Pearson correlations and corresponding regression lines showing the relation between the proportions that infants (top) and mothers (bottom) engaged in transitions from target-to-same-target, target-to-new-target, nothing-to-nothing, target-to-nothing, and nothing-to-target, by session number. Trend lines for each participant show the direction of the relation. Light gray and darker gray shaded areas span the range of session numbers during which different infants learned to crawl and walk, respectively.

To understand more about mothers’ and infants’ interactive behaviors in their environment, we tracked if they changed targets between successive intervals, maintained the same target across successive intervals, went from a target to nothing on the next interval (or the reverse), or did not engage at all with targets for a few intervals. These different types of target transitions were normalized out of the total target transitions possible. Figure 7 displays the developmental trends as regression lines from Pearson’s correlations for all 13 infants and 13 mothers for each types of target transition.

Friedman tests comparing the correlation values of those trend lines between target transition types and sessions revealed significant differences for the infants [χ^2^(4) = 29.846, *p* < 0.0001], but none for the mothers [χ^2^(4) = 4.862, *p* < 0.302]. Infants had positive correlations for target-to-new-target (mean *r* = 0.697) that were significantly different from the negative or near zero correlations of all other target transition categories (all *p*s < 0.001). Thus, over the entire study period, infants not only increased their bouts of interactions, but they also increasingly transitioned to new targets between consecutive time intervals, while all other target transition types either declined or remained about the same over time. The mothers did not reveal significant changes in target transitions over the duration of the study.

**FIGURE 8:**
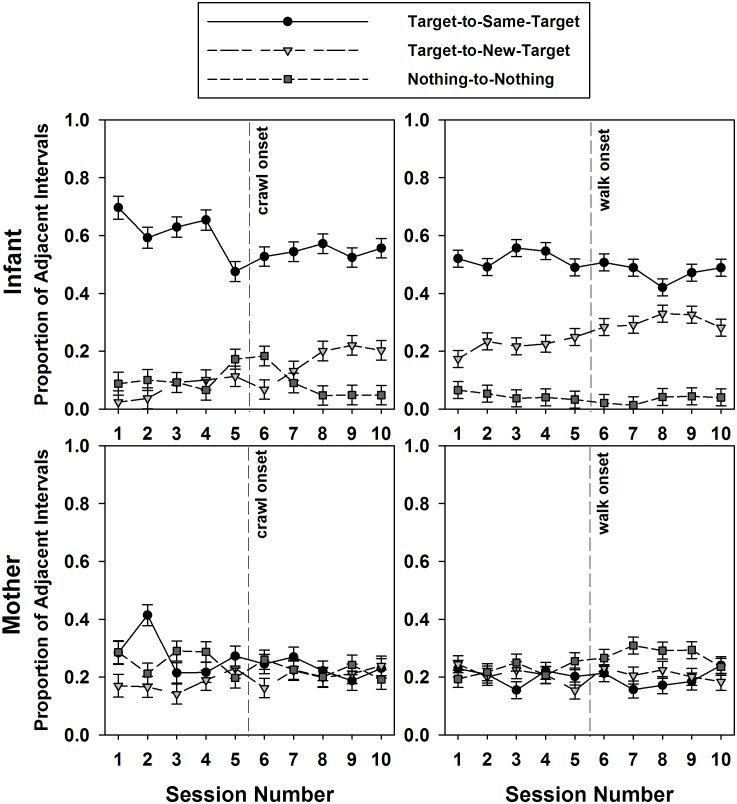
Mean proportions and standard errors of infants’ (top) and mothers’ (bottom) target transitions by type and by session number. The graphs represent the 10-session transition around crawling (left) and walking (right), with vertical lines indicating the onsets of crawling and walking, respectively.

Since little developmental variations were found for the target-to-nothing and nothing-to-target transitions, we ran the GEE analyses using dyad (mother vs. infants), target transition type, and session as predictors only on the three categories of target transitions showing developmental change (i.e., target-to-same-target, target-to-new-target, and nothing-to-nothing, see Figure 8). Around the crawling period, the GEE revealed a main effect of target transition type [Wald χ^2^(2) = 249.354, *p* < 0.0001]. The proportion of successive time intervals in which mothers and infants interacted with the same target was on average significantly higher (41.63%) than the two other target transition types (15.62 and 16.61%, *p*s < 0.0001). However, a significant dyad × transition type interaction [Wald χ^2^(2) = 179.799, *p* < 01.0001] indicated that this effect was mainly driven by the infants. On average, infants interacted with the same target across successive time intervals significantly more (57.71%) than they transitioned to new targets (11.96%) or from nothing-to-nothing (9.37%, all *p*s < 0.0001), while mothers did not show any trend. The GEE also reported a target transition × session interaction [Wald χ^2^(18) = 42.318, *p* < 0.001], and a dyad × target transition × session interaction [Wald χ^2^(18) = 38.112, *p* < 0.004]. Infants’ proportion of successive intervals interacting with the same target declined over this crawling transition period while the proportion of transitions to new targets increased. Again, mothers did not reveal much changes over time.

A GEE analysis on percent intervals of target transitions using the same predictors and the same target transition types over the transition to walking revealed similar trends (Figure 8). Main effects of dyad [Wald χ^2^(1) = 12.618, *p* < 0.0001] and target transition type [Wald χ^2^(2) = 163.543, *p* < 0.0001], and a significant dyad × target transition type interaction [Wald χ^2^(4) = 459.489, *p* < 0.0001] indicated that the target-to-same-target transitions still occurred on average more frequently over successive time intervals than the other two target transition types (34.83% compared to 23.45, 14.53%, all *p*s < 0.0001). However, this was again mainly the case for the infants, who produced on average 49.83% of target-to-same-target transitions compared to 26.15% target-to-new-target transitions and 3.93% of nothing-to-nothing transitions (all *p*s < 0.0001). Mothers did not demonstrate significant differences between target transition types.

In sum, infants produced many target-to-same or target-to-new-target transitions over the observed developmental period compared to their mothers and produced very few target-to-nothing, nothing-to-target, or nothing-to-nothing transitions over successive time intervals. Over time, infants gradually decreased their rate of target-to-same-target transitions and increased their rate of target-to-new-target transitions, suggesting that with the acquisition of mobility, infants explored their environment more widely and interacted with more targets. Mothers did not show much change in their target transitions over time; neither did they display a predominant type of target transition.

### Target Transitions and Posture Changes in Infants

**FIGURE 9:**
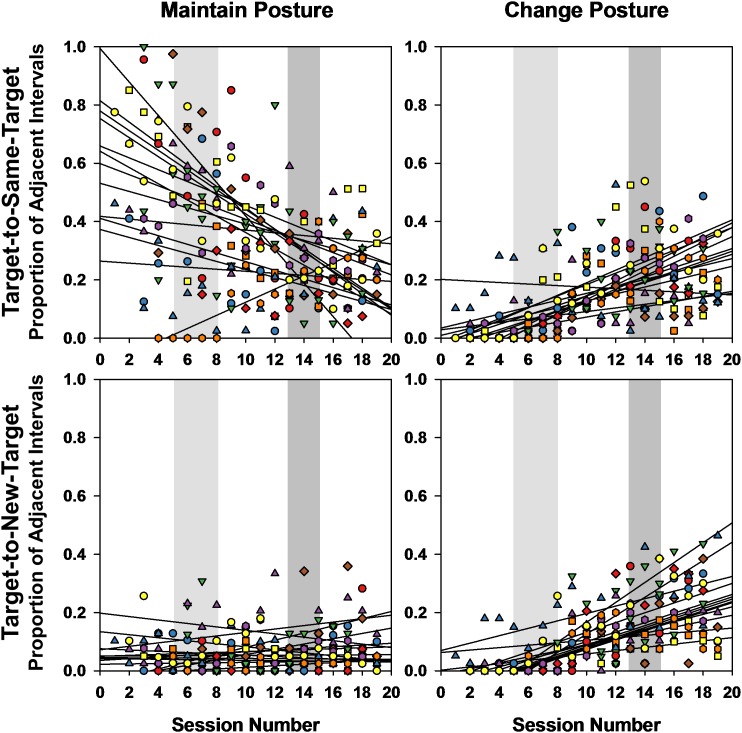
Pearson correlations and corresponding regression lines showing the relation between the proportion of transitions infants engaged in transitions from target-to-same-target (top) and target-to-new-target (bottom), while also maintaining (left) or changing posture (right), by session number. Light gray and darker gray shaded areas span the range of session numbers during which different infants learned to crawl and walk, respectively.

Given that infants were the only ones showing high transitions between same and new targets, we also examined, for the infants only, whether these two types of target transitions corresponded to a change or maintenance of posture over the same successive time intervals. Figure 9 displays regression lines from Pearson’s correlations for each of the 13 infants indicating the developmental trends for maintaining posture vs. changing posture during target-to-same-target transitions and target-to-new-target transitions. The Friedman test on the obtained correlation coefficients revealed that the developmental trends for those four case scenarios were significantly different [χ^2^(3) = 31.985, *p* < 0.0001]. In target-to-same-target transition intervals, posture maintenance declined over time (mean *r* = -0.551) posture changes increased (mean *r* = 0.594, *p* < 0.002). For the intervals of target-to-new-target transitions, posture maintenance did not change over time (mean *r* = 0.098), but posture changes also increased (mean *r* = 0.691, *p* < 0.001).

**FIGURE 10:**
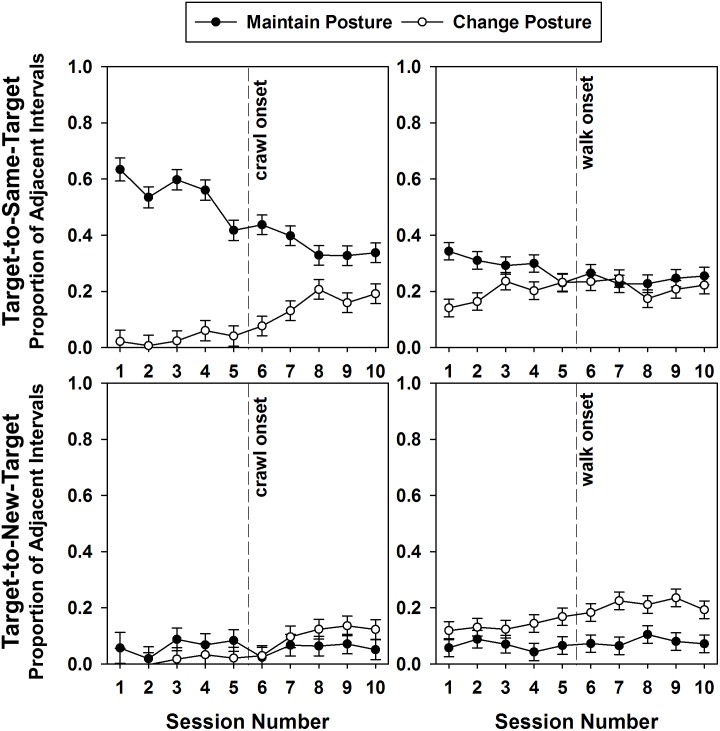
Mean proportions and standard errors of infants’ target-to-same-target (top) and target-to-new-target (bottom) transitions as a function of corresponding posture transitions by session number. The graphs represent the 10-session transition to crawling (left) and walking (right), with the vertical lines indicating the onsets of crawling and walking, respectively.

A GEE on these percentage intervals of posture/target changes using the type of target transition with type of posture change and session as predictors around the emergence of crawling (Figure 10) revealed a main effect of posture change/target transition [Wald χ^2^(3) = 400.703, *p* < 0.0001] and a significant posture change/target transition × crawling session interaction [Wald χ^2^(27) = 89.543, *p* < 0.0001]. Posture maintenance during target-to-same-target transition was the behavior most highly performed by the infants (45.73%), and was on average significantly different from all three other posture/target transition combinations (range = 5.75–9.19%, all *p*s < 0.0001). However, the interaction indicated that posture maintenance during target-to-same-target transitions declined significantly over the 10-session crawling period (*p*s from session 7 < 0.003), while posture changes increased. For the target-to-new-target transitions, posture maintenance and posture change did not occur much over this crawling period and represented less than 20% of the successive intervals.

A GEE on this same variable using the same predictors over the walking transition period (Figure 10) only returned a main effect of posture change/target transition [Wald χ^2^(3) = 99.12, *p* < 0.0001]. The interaction with sessions of walking did not reach significance [Wald χ^2^(27) = 38.555, *p* < 0.07]. Posture maintenance during same-target interval transitions was again on average more represented (26.96%) than the other posture change/target transition combinations (7.18, 17.32, 20.6%, all *p*s < 0.008).

Thus, the early period, corresponding to the transition to crawling presented the greatest developmental change in posture during target transitions. Change in posture increased whereas posture maintenance declined, especially during same-target transitions.

## Discussion

A growing body of literature underscored the importance of infants’ action experience for their understanding of the world (e.g., Sheya and Smith, 2010; Soska et al., 2010). Developmental researchers have also examined the relation between infants’ sense of agency, locomotor experience, and environmental characteristics in shaping infants’ actions (e.g., Dosso and Boudreau, 2014), and how their actions both influence and are influenced by interactions with their caregivers (e.g., Karasik et al., 2014). Prior findings from this dataset revealed that the rate of posture changes was related to the number of bouts of interaction infants performed during free play, but this was not true for mothers (Thurman and Corbetta, 2017). The current study extended this work, and investigated how mothers and infants adopted various postures during play, how they used postures to interact with targets in their environment, and how infants’ repertoire of postural skills expanded over the course of locomotor development.

As one would expect, infants broadened postural diversity and interactive behaviors during object interaction as they gained locomotor skills over time, but interestingly, as they did so, they also reorganized the way they used prior occurring postures to manipulate their environment. For example, sitting which was mostly used for fine motor manipulation during the crawling transition period, started to be used increasingly more for passive holding during the walking transition period, and at that same time, kneeling/squatting became the preferred postures for fine motor manipulation. Regardless, infants engaged in targeted behavior most of the time and interacted with the same target from interval to interval more frequently than they changed to a new target. They also tended to maintain the same posture when attending to the same target across intervals despite developing a growing range of postures. Mothers on the other hand remained passive or minimally engaged most of the time, even though they had the freedom to move about the room and interact with their infants. With mobility, infants’ bodies seemed to become tools for exploration, allowing for a growing diversification of their behavior and interactions with their surroundings, interspersed with moments of posture maintenance with a same target.

### Infants’ and Mothers’ Interactions and Postures

Mothers spent much less time interacting with targets compared to their infants, and this did not change very much longitudinally as their infants acquired locomotor skills. This finding may be consistent with prior work in home settings, which has shown that mothers tend to respond similarly to their infants over time (e.g., Masur and Turner, 2001), and mothers often arrange play spaces for self-initiated infant play (Pierce, 2000).

While mothers’ interactions seemed to be more predictable and stable, infants’ interactions developed and reorganized in concert with their growing postural options (Thelen and Smith, 1994). The early fine motor manipulations with objects performed during sitting, progressively morphed into holding patterns later in the study. Early sitting frees the hands to manipulate objects and has been associated with fine haptic explorations and differential functioning of the hands (Rochat, 1989). But as infants increasingly varied their postures and were free to play, kneeling and squatting emerged as the new postures for fine motor manipulation of targets along with passive involvement with targets. Indeed, when infants stop moving around the room and want to examine an object, kneeling or squatting are the next easiest postures to produce, and recent research suggests squatting postures even enhance postural control (Bril, 2018). Sitting and kneeling/squatting occurring during the later developmental period, that gave rise to more passive involvement, may at that point have become more transitional postures (than standing postures). Infants can adopt those postures for short moments on their way to their next object destination. A more in-depth examination of these data in future studies will allow us to address these questions more readily.

As infants began to stand, they revealed the highest rate of passive interactions with targets. Standing, initially, is a very unstable posture. Holding an object helps stabilize the upright posture (Claxton et al., 2013), but it may be that when standing, infants are busy maintaining balance, which may temporarily affect their ability to perform detailed object manipulations or gross motor activity on objects. Metcalfe and Clark (2000) have shown that when infants keep their hand on a surface while standing, they are using the surface contact as source of postural stabilization, but also as a way to explore their own developing postural coordination. Consistent with that study, we found that gross motor activity, which was seldom represented in the early period, progressively increased during the later kneeling/squatting and standing postures. Here also, future research could investigate more closely how infants learn to control their bodies and postures in relation to acting on their environments over time.

This work supports previous claims that postural development and postural control both play important roles in the execution of skilled and target-directed actions in infancy (Rochat and Bullinger, 1994). At every stage throughout development, as infants learn to sit, kneel, crawl, and walk, they learn information about their body’s resources and action capabilities, and this greatly affects their ability to interact with the resources and opportunities provided in the environment (Gibson, 1988; Rochat and Bullinger, 1994; Adolph, 2008). Importantly, in developmental pathway approaches, cumulative change builds complexity in developmental systems, and rudimentary exploratory skills lay a foundation for which later-appearing skills can be built upon (Thelen and Smith, 1994; Smith, 2013). For example, Bornstein et al. (2013) discovered that early motor and exploratory behaviors in infancy lay a foundation for future intellectual functioning and academic achievement in childhood. This is because opportunities for interacting with the environment, which are promoted by motor and exploratory behaviors, can lead to crucial learning opportunities for the infant. In our analyses we have not examined whether learning opportunities were also provided by the mothers as infants and mothers interacted with each other. We know mothers often scaffold infants’ play, which leads infants to display more advanced functional play during joint attention moments (Bigelow et al., 2004). This is a question we are planning to examine in future analyses.

### Postural Development and Target-Directedness

Investigating self-directed infant locomotion provides some insight into the information that infants select from their environments (Dosso and Boudreau, 2014). Our data show that as infants develop locomotion, infants not only produced an increasingly higher rate of targeted behaviors, but they also transitioned to new targets in addition to maintaining interactions with the same target. Others have shown infants spend a great deal of time interacting with objects in their surroundings (e.g., Cole et al., 2016; Hoch et al., 2018), but the way in which infants arrive at those targets has been contested recently. Prior characterizations of infant sensorimotor and functional play patterns referred to Piaget’s early descriptions of infant behavior, which describe infant play as more intentional and goal-directed, such that infants tend to seek out certain kinds of stimulation (Burghardt, 2006). Recent work by Cole et al. (2016) suggests otherwise. They investigated whether bouts of infant walking ended with infants making contact with toys. They found that when infants ended a bout of walking, they most often stopped in the middle of the floor, and many interactions with objects occurred after infants were already in motion. They concluded that infant’s behavior is not goal-directed in the sense that an infant may see a goal in the distance and then travel to it. Instead, because infants cover so much ground, they happen upon opportunities for interaction along the way and while already in motion.

Further, the presence of toys seems to elicit different patterns of locomotor exploration in infants. Recent research compared infants’ exploration patterns in toy-filled vs. empty rooms. Although infants traveled similar distances across the two conditions and took about the same number of steps, in the toy-filled room, infants showed greater spread of exploration compared to infants in the empty room. These differences may be related to how infants interacted with locomotor toys, which are designed to be rolled or carried (Hoch et al., 2018).

Our data, which considered all infant postures and movements, and not just those that occurred in bouts of walking, similarly suggest that infants’ behaviors are highly target-directed. But, we did find particularly in the later period, that infants increasingly involve their whole bodies when interacting with objects, changing body posture when remaining with similar targets or switching to new ones.

### Implications

An infant’s ability to learn new things about their environment is strongly related to exploratory skills that arise with locomotion (Bornstein et al., 2013). Here, we have shown throughout locomotor development, that infants gain more postural options, which in turn affect how they use their bodies and postures to interact with and transition between targets in their environment. Mobility impairments such as Down syndrome and cerebral palsy in infancy can severely delay or completely prevent mobility (Cobo-Lewis et al., 1996; Ghazi et al., 2016), which in turn, can reduce the range of opportunities for interaction that infants possess.

Furthermore, more general motor impairments can also affect how infants use their posture and manipulate objects. In a study by Nickel et al. (2013), infants who later received an autism diagnosis had previously shown slower development of sitting and standing postures, and exhibited fewer posture changes during play. Delays in postural and motor development such as those seen in infants who are at high risk for autism limit opportunities infants have to explore objects and their surroundings. This early disruption in interaction patterns can set the stage for further atypical experiences both within the realm of postural and locomotor skills, but also cognitive development and social interactions (Thelen, 2004).

## Conclusion

Our observations were done in a free-play session, where there were no instructions as to which toys participants should choose in their activities. Furthermore, in order to provide developmentally appropriate toys for participants, we occasionally changed out a small number of objects as infants progressed through motor skills. This may have affected the likelihood that infants would have engaged in particular interactive behaviors in a given session. However, it was our intent to capture variations in behaviors in our free-play format. Varied opportunities for interactions could occur at each session whether the objects in the room were identical or not. We are confident that our findings are unaffected by the small variations in toy selection at any session, as 68% of infants’ target interactions were with items that remained in the room throughout the whole study.

The current study utilized an extensive longitudinal approach to investigate infants’ and mothers’ use of posture for playful interaction with their environments both within sessions and across infant locomotor development. We discovered that with locomotor development, infants’ interactions in their environments changed depending on which postures they adopted in the moment, the range of postures they displayed, and how they used their postures to transition between targets. Mothers, however, remained largely inactive and did not alter significantly patterns of interactions as their infants did. Our approach provided evidence to further support the notion that infants’ use of posture is a dynamic and essential part of their action repertoire during exploration. Our observations were limited to 15 s interval time sampling over just 10 min free-play sessions. While one could argue that this is not sufficient to capture these developmental dynamics, our observations show that they map logically on expected patterns of development.

## Ethics Statement

This study was carried out in accordance with the recommendations of the Institutional Review Board at the University of Tennessee - Knoxville with written informed consent from all parents of the infants. All parents gave written informed consent for their infants in accordance with the Declaration of Helsinki. The protocol was approved by the Institutional Review Board at the University of Tennessee - Knoxville.

## Author Contributions

This work is part of ST dissertation project. DC was her advisor. ST had a primary role in designing and collecting the data and took as well the lead in coding and analyzing the data. DC provided guidance in planning the design and data collection, and provided inputs throughout the coding and data analysis process. ST and DC contributed to the writing of this manuscript.

## Conflict of Interest Statement

The authors declare that the research was conducted in the absence of any commercial or financial relationships that could be construed as a potential conflict of interest.
